# The diagnostic application of targeted re-sequencing in Korean patients with retinitis pigmentosa

**DOI:** 10.1186/s12864-015-1723-x

**Published:** 2015-07-09

**Authors:** Chang-Ki Yoon, Nayoung K. D. Kim, Je-Gun Joung, Joo Young Shin, Jung Hyun Park, Hye-Hyun Eum, Hae-ock Lee, Woong-Yang Park, Hyeong Gon Yu

**Affiliations:** Department of Ophthalmology, Seoul National University College of Medicine, Seoul, Korea; Department of Biomedical Sciences, Seoul National University Graduate School, Seoul, Korea; Department of Molecular Cell Biology, Sungkyunkwan University School of Medicine, Seoul, Korea; Samsung Genome Institute, Samsung Medical Center, Seoul, Korea; Department of Ophthalmology, Seoul Paik Hospital, Inje University, Seoul, Korea

**Keywords:** Retinitis pigmentosa, Targeted re-sequencing, Genetic diagnosis, Familial case, Sporadic case

## Abstract

**Background:**

Identification of the causative genes of retinitis pigmentosa (RP) is important for the clinical care of patients with RP. However, a comprehensive genetic study has not been performed in Korean RP patients. Moreover, the genetic heterogeneity found in sensorineural genetic disorders makes identification of pathogenic mutations challenging. Therefore, high throughput genetic testing using massively parallel sequencing is needed.

**Results:**

Sixty-two Korean patients with nonsyndromic RP (46 patients from 18 families and 16 simplex cases) who consented to molecular genetic testing were recruited in this study and targeted exome sequencing was applied on 53 RP-related genes. Causal variants were characterised by selecting exonic and splicing variants, selecting variants with low allele frequency (below 1 %), and discarding the remaining variants with quality below 20. The variants were additionally confirmed by an inheritance pattern and cosegregation test of the families, and the rest of the variants were prioritised using *in-silico* prediction tools. Finally, causal variants were detected from 10 of 18 familial cases (55.5 %) and 7 of 16 simplex cases (43.7 %) in total. Novel variants were detected in 13 of 20 (65 %) candidate variants. Compound heterozygous variants were found in four of 7 simplex cases.

**Conclusion:**

Panel-based targeted re-sequencing can be used as an effective molecular diagnostic tool for RP.

**Electronic supplementary material:**

The online version of this article (doi:10.1186/s12864-015-1723-x) contains supplementary material, which is available to authorized users.

## Background

Retinitis pigmentosa (RP) is a group of hereditary retinal diseases characterised by progressive loss of rod and cone photoreceptor cells. It is the most common hereditary retinal disease with a frequency of approximately 1 in 3000–4000 people, with an estimated total of 1.5 million people affected worldwide [1, 2]. Patients with RP usually present with a loss of night vision and complain of progressive constriction of the visual field. Eventually, central vision is affected and worsens. Clinical features such as age of onset and rate of progression vary greatly between individuals.

The identification of causative genetic variants is expected to be the starting point of RP treatment. Genetic counselling and gene therapy or other patient-specific treatment options can be suggested based on genetic background. In addition to treating the disease, molecular diagnosis is helpful for informing the differential diagnosis. Hereditary retinal dystrophies have a slowly progressive nature and complete clinical features may not be present at the time of examination. Therefore, many other hereditary retinal dystrophies may be confused with RP at certain phases of disease progression.

However, the genetic heterogeneity of RP is a hurdle for the easy application of molecular diagnosis. At the time of September 2011, 53 genes and 7 mapped loci are known to be associated with nonsyndromic RP [3] (RetNet). These genes show considerable phenotypic variability and multiple inherited retinal disease shares certain causative genes with RP [4]. Because of this phenotypic heterogeneity and confusion between genotypic and phenotypic correlations, the clinical features of individual patients provide few clues to narrow down the list of candidate genes [2, 5]. Therefore, these genes and loci should be screened simultaneously.

Massively parallel sequencing, also called next-generation sequencing (NGS) is a recent technical breakthrough in genetics [6, 7]. NGS-based high-throughput sequencing even makes whole-genome sequencing available in a single facility, which would have required tremendous cost and time in the past. RP can be a good target for massively parallel sequencing as an appropriate number of candidate genes are known for RP. Therefore, this study attempts to detect candidate variants in RP by applying a targeted enrichment and re-sequencing strategy.

## Results

### Quality validation of sequencing

A total of 217.5 kbp of targeting and flanking regions from 53 RP-related genes and 748 RP-related exons were captured; 97.1 % of the 2.89 M reads obtained on average per individual were properly paired and mapped (Additional file 1: Table S1). The average read depth was 146.2 ± 26.2, and 98.6 % and 96.5 % of the captured target exons showed more than 1X and 10X coverage, respectively (Additional file 1: Table S1).

### Detection of variants in familial RP

Strong candidate variants of RP were identified in 10 families (Table 1). Among 18 families, 10 had an autosomal dominant, five had an autosomal recessive and three had an X-linked inheritance pattern. Briefly, five known pathogenic variants (c.421-1G > A in *PRPF31*, p.P347L in *RHO*, p.A153V in *KLHL7*, p.Y178C in *RHO*, p.R354X in *PRPF31 w*ere detected [8–13]. The known variant, pC3416G in *USH2A*, was initially detected, but the other variant, p.G4647R in the XF4 family under the inheritance pattern of autosomal recessive (compound heterozygote), was predicted as non-pathogenic. Therefore, the XF4 family was excluded. The other five candidate variants in *RP1*, *RP2* and *TOPORS* have not been reported in dbSNP and were co-segregated in the family members.

**Table 1:** Strong candidate variants in familiar cases

Family	Inheritance	Gene	Genotype	Chr	Exon	nucleotide	amino acid	Mutation Type	Reference	EVS	In-house	Class
F03	AD	RP1	Hetero	8	4	c.1455 T > G	p.Y485X	nonsense	novel	-	-	II
F04	X-L	RP2	Hemi	X	2	c.340 T > C	p.C114R	nonsynonymous	novel	-	-	II
F06	AD	RP1	Hetero	8	4	c.2296C > T	p.Q766X	nonsense	novel	-	-	II
F07	AD	PRPF31	Hetero	19	6	c.421-1G > A		splicing	Xia et al. [11]	-	-	I
F09	AD	RHO	Hetero	3	5	c.1040C > T	p.P347L	nonsynonymous	rs29001566	-	-	I
F10	AD	KLHL7	Hetero	7	5	c.458C > T	p.A153V	nonsynonymous	rs137853113	-	-	I
F12	X-L	RP2	Hemi	X	2	c.560_561delGC	p.Ser187fs	Frameshift deletion	novel	-	-	II
F13	AD	RHO	Hetero	3	3	c.533A > G	p.Y178C	nonsynonymous	rs104893776	-	-	I
XF1	AD	TOPORS	Hetero	9	2	c.2344C > T	p.R782X	nonsense	novel	-	0.0052083	II
XF3	AD	PRPF31	Hetero	19	10	c.1060C > T	p.R354X	nonsense	Sullivan et al. [12]	-	-	I

*Chr*: chromosome; Homo: homozygous; Hetero: heterozygous; Hemi: hemizygous; AD: Autosomal Dominant; X-L X-linked

In-house: Korean normal reference consisting of 192 exomes

The 10 variants were comprised of four nonsynonymous variants, four nonsense mutations, one frameshift deletion, and one splicing variant. All the nonsense mutations (p.Y485X in *RP1*, p.Q766X in *RP1*, p.R782X in *TOPORS*, and p.R354X in *PRPF31*) were located at the upstream exons (exon 4 of 4, exon 3 of 4, exon 2 of 3 and exon 10 of 16 respectively), which might lead to nonsense-mediated decay of mRNA. A frameshift variant (p. Ser187fs in *RP2*) was also located at the 5′ upstream exon, and all the nonsense and frameshift variants were classified as possible pathogenic variants (category II).

The clinical features were relatively severe in patients having X-linked variants, F04 (p.C114R in *RP2*) and F12 (p.Ser187fs in *RP2*) (Additional file 1: Table S2). The affected family members experienced early onset of symptoms and presented with rapid progression. In the F04 family, the proband (VI-1) was a 6-year-old male whose visual acuity was 20/200 (OD) and 20/1000 (OS). The visual field was mildly constricted using a Goldmann perimetry V4 target, and the rod responses were extinguished and photopic responses were severely decreased on the electroretinogram. The photoreceptor inner segment and outer segment junction was not detectable using OCT (optical coherence tomography). The patient’s grandfather (II-1) had no light perception in both eyes. Fundus examination and OCT scan showed a severely degenerative retina. For family F12, a possible pathogenic missense variant was found in the X-linked *RP2* gene. Hemizygous male patients (II-5, II-16 and III-2) showed poor visual acuity at less than counting fingers. The retina was totally degenerated such that the retina and choroid were not distinguishable on OCT. Scoptopic and photopic electroretinogram (ERG) were all extinguished. Female patients of the F12 family (II-8, II-13, III-11 and III-20) showed variable penetrance. Three of them (II-13, III-11 and III-20) were confirmed to have heterozygous variants. Two female patients (III-11 and III-20) had mild peripheral pigmentary deposits and decreased rod and cone amplitude on electroretinography. Two other female patients (II-8 and II-13) showed complete expression of RP. They had visual acuity limited to light perception in both eyes, typical bony spicule pigment deposits, attenuated retinal vessels. Scoptopic and photopic ERG amplitude were also abolished.

F06 (p.Q766X in *RP1*) showed a milder phenotype. In family F06, all patients experienced their first symptoms in their 30s. Two patients older than age 50 (II-3, 75 years old; III-2, 54 years old) had visual loss, while the other patients (III-6, III-10 and III-13) had normal vision. Patient III-6 and III-10 showed a normal macula on OCT and a mildly constricted visual field. The clinical features of the patients and pedigrees are summarised in Additional file 1: Table S2 and Figure S1. Fundus photograph and OCT are presented in Additional file 1: Figure S2.

### Detection of variants in simplex RP

The possible pathogenic variants were also identified in 7 of 16 patients with simplex RP (43.7 %) (Table 2). These patients denied a familial history of RP or related hereditary disorders. The inheritance pattern of candidate genes was based on the sequencing results (Table 2). Initially four known pathogenic variants, p.H278Y in *PDE6B*, p.Y2935X and p.G2186E in *EYS*, and p.C3416G in *USH2A*, were detected. [13–16]. However a possible second variant in *EYS* (patient 435) and *USH2A* (patient 450) was not found, p.Y2935X (*EYS*) and p.C3416G (*USH2A*) were not included in final results. Eight causal candidate variants were newly identified. Overall, ten possible pathogenic variants were detected. They were comprised of six nonsynonymous, three nonsense, and one frameshift insertion variants. Compound heterozygous variants (*in trans*) were detected in five patients (*PDE6B* in 436 and 445, *EYS* in 439 and 440, *USH2A* in 438). The same frameshift variant of *EYS* (p.S1653fs) was identified in two unrelated patients with simplex RP (439 and 440).

**Table 2:** Strong candidate variants in sporadic cases

No	Inheritance*	Gene	Genotype	Chr	Exon	nucleotide	amino acid	Mutation Type	Reference	EVS	In-house	Class
430	AD	PRPF31	Hetero	19	1	c.310G > A	p.E104K	nonsynonymous	Novel	-	-	II
432	AD	PRPH2	Hetero	6	1	c.380A > G	p.E127G	nonsynonymous	Novel	-	-	II
436	AR	PDE6B	Compound hetero	4	8	c.832C > T	p.H278Y	nonsynonymous	rs121918581	-	-	Ι
					1	c.32G > A	p.W11X	nonsense	Novel	-	-	II
438	AR	USH2A	Compound hetero	1	42	c.8885 T > G	p.L2962R	nonsynonymous	Novel	-	-	II
					18	c.4460G > A	p.W1487X	nonsense	Novel	-	-	II
439	AR	EYS	Compound hetero	6	8	c.1750G > T	p.E584X	nonsense	Novel	-	-	II
					26	c.4958_4959insA	p.S1653fs	frameshift insertion	Novel	-	-	II
440	AR	EYS	Compound hetero	6	29	c.6557G > A	p.G2186E	nonsynonymous	10 Littink	-	-	Ι
					26	c.4958_4959insA	p.S1653fs	frameshift insertion	Novel	-	-	II
445	AR	PDE6B	Compound hetero	4	8	c.832C > T	p.H278Y	nonsynonymous	rs121918581	-	-	I
					8	c.767 T > A	p.I256N	nonsynonymous	Novel	-	-	II

*Inheritance is not inferred from pedigrees of the patients. These patients stated that there is no affected individual in their family tree other than indexed patients. Inheritance pattern described the results suggested by sequencing data

In-house: Korean normal reference consisting of 192 exomes

*Chr* chromosome; *AD* Autosomal dominant; *AR* Autosomal recessive; *X-L* X-linked

Regarding the clinical feature, patient 430 with the *PRPF31* mutation (p.E104K) showed a mild clinical phenotype. Although he was 42 years old, his visual acuity was 20/20 in both eyes, but a paracentral scotoma was detected in the visual field test. The electroretinogram showed a small decrease only in rod response. Most of the photoreceptor inner segment and outer segment junction (5 mm in the horizontal scan) was observed intact using an OCT macula scan (Additional file 1: Table S3). Fundus photograph and OCT are presented in Additional file 1: Figure S2.

## Discussion

In this study, targeted exome capture and massively parallel sequencing (MPS) was implemented for genetic diagnosis of RP. Strong candidate variants were identified in 10 (55.5 %) of 18 families and 7 (43.7 %) of 16 sporadic patients. The overall detection rate was 50 %. This method seems to be highly efficient and cost effective in comparison with PCR amplicon sequencing and other conventional methods such as microarray analysis. It is certainly much higher than the 7–16 % detection rate of microarray genotyping [17–21]. A previous genetic analysis of Korean RP patients using microarrays revealed a causative mutation in 26 out of 336 patients (2 %) [19]. The subjects of this microarray study were different from the current study. Patients from the F09 and F13 families had also been screened for detection of the *RHO* mutation by using direct sequencing in a previous study [22]. The current study analysed 53 target genes and showed exactly the same results as the direct sequencing of the *RHO* gene for these particular individuals, thereby supporting the accuracy and efficiency of the current strategy. The current results are also comparable with other RP genetic analysis using targeted resequencing, in which the detection rate ranged from 36 to 82 % [23–28]. Unbiased sequencing, including whole-genome or exome sequencing, can provide comprehensive genetic data that reveal novel causal genes of RP such as *NEK2*, *HK1* and *MVK*. However the sequencing and data processing burden is still the bottleneck for widespread use of unbiased sequencing. Moreover, detection rates using whole exome sequencing have not proven to be significantly better than targeted resequencing as previously expected [29]. This also supports the usefulness of targeted exome sequencing in RP genetic screening.

Twenty possible causal variants were detected from 10 genes. Of these, 13 variants (65 %) were novel and this proportion is comparable with recent works reporting novel allele rates of 62–68 % [25–27, 29]. The considerable number of novel variants further emphasises the use of MPS as a reasonable tool for RP molecular diagnosis. The *PRPF31* mutation (17.6 %) is most frequently found in current study, followed by mutations in *EYS*, *PDE6B*, *RHO*, *RP1*, and *RP2* (11.8 % respectively). *PRPF31* (30 %), *RHO* (20 %), and *RP1* (20 %) were frequently affected genes in autosomal dominant RP (adRP), whereas *EYS* (40 %) and *PDE6B* (40 %) were frequently affected genes for autosomal recessive RP (arRP). Despite the limitation of extending this result to the general distribution of RP mutations in Koreans, frequently affected genes are similar to those reported in recent works as well as previous reports by Hartong et al. [2, 23, 27, 29]. Possible causal genes responsible for simplex cases had primarily autosomal recessive inheritance (5 out of 7 cases, 71 %) as suggested previously [1, 2]. It could not be confirmed whether causal alleles were *de novo* because segregation analysis was not able to be performed in simplex cases.

In X-linked RP, the *RPGR* gene is thought to be the most common causal gene, accounting for over 70 % of cases [30]. Although the *RPGR* gene was included in the capture library of the present study, the candidate causal variant could not be found. ORF15 is a mutation hotspot of *RPGR*, where roughly two thirds of disease-causing mutations are found [30]. This repetitive purine-rich ORF15 region is rarely covered by next-generation sequencing. Therefore, the *RPGR* mutation could have been missed and, instead, only the RP2 variants detected. A missense variant (p.C114R) in family F04 and a truncating variant (p.S187Tfs*31) in family F12 was identified. The missense variant (p.C114R) was located in the conserved tubulin binding cofactor C (TBCC) domain and was evolutionarily conserved. Interestingly, family F12 showed an autosomal dominant pattern without male to male transmission. Unlike the expected pattern of an X-linked disease affecting only males, recent studies have reported that *RP2* and *RPGR* mutation cause variable RP phenotypes in heterozygous female patients.[30–32] Furthermore, it is reported that mutations in *RP2* and *RPGR* account for 8.5 % of patients with RP in provisional autosomal dominant families [33].

*PRPF31* variants were identified in two families and one simplex RP patient. One splicing site variation (c.421-1G > A in family F07) and truncating variant (p.R354X in family XF3) were known pathogenic variants [11, 12]. Haploinsufficiency from a null allele rather than gain of function is assumed to be the major mechanism of *PRPF31* mutation-derived RP [34]. However, missense variants also have been suggested as possibly pathogenic in several reports [34–37]. A missense variant (p.E104K) was also detected in a simplex RP patient (430) in the current study. This substitution is located at the highly conserved NOSIK domain and this sequence is evolutionarily conserved.

An *EYS* variant was identified in two simplex patients. The same frameshift insertion variant (p.S1653Kfs*) was identified in two unrelated patients. As a second candidate allele, 439 had a premature truncating variant (p.E584X) and 440 had a missense variant (p.G2186E). Nonsense mediated decay from a heterozygous compound truncating mutation is assumed to be a possible pathogenic mechanism in 439. The missense variant (p.G2186E) in patient 440 was located in the highly conserved Laminin G domain. Interestingly, this mutation was previously reported in a patient with Korean ancestry [16]. A frameshift variant (p.S1653Kfs*) and missense variant (p.G2186E) were recurrent in a relatively small cohort and it is assumed that these could be founder variants in Koreans. Iwanami et al. suggested that patients having a homozygous or compound heterozygous truncating mutation of *EYS* show a more severe decline of visual function than patients having only one allele of the truncating variant [15]. However, both cases (439 and 440) did not show any difference in progression in the current study independent from number of truncating variants.

A known pathogenic missense variant (p.H278Y) of *PDE6B* was detected in two unrelated patients (436 and 445). A truncating variant (p.W11X) was found in patient 436 as a second variant and a novel missense variant (p.I256N) was detected in patient 445. This missense variant is located in the highly conserved GAF domain. Loss of function of cGMP phosphoodiesterase activity is a possible mechanism in these two patients.

Just one possible pathogenic allele was found in the XF4 family and simplex patients 433, 435 and 450. (Data not shown in Tables 1 and 2) The identified genes in these patients have an autosomal recessive inheritance pattern. Known pathogenic variants (p.C3416G of *USH2A* in XF4 and 450, p.Y2935X of *EYS* in 435) were found in just one allele. As a second pathogenic candadate, missense variants p.T2465S of *EYS* and p.V2228E of *USH2A* were detected and predicted to be pathogenic in patient 435 and 450, respectively. (Additional file 1: Table S4) These were relatively common variants (more than 1 %) in the genomic data of 1000 Asians. Considering that the counter allele, p.Y2935X and p.C3416G, is rare and pathogenic, these candidate variants may also be pathogenic alleles. Regarding other cases in the current study (XF4 and 433), a second pathogenic allele could not be identified. Meaningful second variants may not have been identified because they were large indels or located in an untranslated region. Alternatively, detected pathogenic variants might be accidental carriers and causal variants are located in other genes. Accidental carrier mutations are increasingly being reported as the use of multigenic screening with MPS increases [23, 27]. Although the current study revealed a possible causative variant in half of cases, obviously a candidate causal allele could not be found in the other half of patients. Since the target library was designed, three more causative genes have been added to the RP gene list. Unknown causal genes for RP can still exist and including these targets would increase the detection yield. However, whole exome sequencing or the use of an extended target library of over 100 genes has not revealed a higher detection rate [25, 29, 38]. Unbiased sequencing not only poses an enormous data processing burden, but also impedes deep sequencing. Covering the non-coding exons of target genes and complete coverage of the target may be better way to increase diagnostic yield rather than extending the target library. Eisenberg et al. reported high detection rates using high coverage, copy number variance (CNV) analysis and sequencing the 5′ UTR [27]. Xu et al. reported that complete coverage using deep sequencing and PCR amplicon for less covered regions yielded an 82 % detection rate [23]. Although a ≥ 10X sequencing depth was reached in 96 % of the sequenced region, 4 % had a less than 10X depth and 1.4 % was not covered (Additional file 1: Table S1). GC-rich regions or highly repetitive regions are usually under-captured by MPS. The mutational hotspot, ORF15 of *RPGR*, is one of those regions. Complete coverage assisted by direct sequencing and deep sequencing is needed to raise the causative variant detection yield.

Identifying a causal mutation is the starting point of RP treatment, followed by proper genetic counselling and prognostic data. For instance, patients can be informed that they have a certain mutation, which puts them at risk for rapid progression of RP, and aggressive therapy can be initiated on the basis of confirmative genetic data, although there are limited treatment options available to help RP patients as of yet. Gene therapy, one of the possible treatment options for RP, is largely dependent on genetic data, indicating that identifying the causal mutation will become an increasingly important step in RP treatment.

## Conclusion

Using the targeted re-sequencing of known genes for RP, pathogenic and possibly pathogenic variants were identified in more than the half of patients and families. This strategy will become an increasingly efficient and cost-effective molecular diagnostic test for retinitis pigmentosa.

## Methods

### Patients

Sixty-two Korean patients with RP were selected for the RP cohort. This cohort consisted of 46 patients from 18 families and 16 patients with simplex RP. All the patients had nonsyndromic RP. Comprehensive ocular examinations, including visual acuity assessment, slit-lamp examination, fundus examination, electroretinograms, Goldmann Perimetry (Haag-Streit, Bern, Switzerland) and optical coherence tomography (Carl Zeiss Meditec Inc., Dublin, CA, USA) were used for clinical diagnosis. RP was diagnosed when the patients had the typical retinal appearance of RP and had attenuated or abolished rod and cone signal on electroretinography. Bony spicule-like pigment deposits, retinal vessel attenuation and optic disc pallor were included in the characterisation of typical retinal features of RP. It was assumed that the mode of inheritance was autosomal dominant when a patient appeared in every generation of the pedigree, and when affected fathers and mothers transmitted the phenotype to both sons and daughters. Autosomal recessive patterns were suspected when a phenotype appeared in the male and female progeny of unaffected parents. X-linked recessive inheritance was suspected when affected males passed the condition on to all of their daughters, but to none of their sons, and female patients married to unaffected males passed the condition on to half of their sons and daughters. Written informed consent was obtained from all patients before they were enrolled in the study. This study followed the tenets of the declaration of Helsinki (Edinburgh, 2000) and was approved by the Institutional Review Board of Seoul National University Hospital.

### Targeted exome sequencing of RP-related genes

Customised baits were designed to capture all exons of the 53 genes known to be associated with RP at the time of panel design. (Table 3, Roche NimbleGen Inc, Madison, WI). Genomic DNA from 62 patients in total was extracted from peripheral blood as described previously [39] and was sequenced using the Genome Analyzer II. Sequencing reads were aligned to the UCSC hg19 reference genome using BWA-0.6.1 with default settings. Duplications were removed via Picard v1.93, and local realignment was done by GATK v2.4-7. Variants were identified by the Unified Genotyper from GATK for the SNVs and indels. ANNOVAR was used to annotate the variants. Coverage of TES data was calculated by the ‘Depth of Coverage’ module from GATK. Sanger sequencing for candidate variants was performed using specific primers for each exon as demonstrated.

**Table 3:** 53 RP-related genes selected for targeted resequencing

Gene	Inheritance	RefSeq	Cytogenetic Loci	Exon Count	Description
ABCA4	AR/AD	NM_000350	1p22.1	50	ATP-binding cassette, sub-family A (ABC1), member 4 (ABCA4)
ARL6	AR	NM_177976	3q11.2	9	ADP-ribosylation factor-like 6 (ARL6), transcript variant 2
BEST1	AD	NM_001139443	11q12.3	9	bestrophin 1 (BEST1), transcript variant 2
C2ORF71	AR	NM_001029883	2p23.2	2	chromosome 2 open reading frame 71 (C2orf71)
CA4	AD	NM_000717	17q23.1	8	carbonic anhydrase IV (CA4)
CERKL	AR	NM_201548	2q31.3	13	ceramide kinase-like (CERKL), transcript variant 1
CLRN1	AR	NM_001195794	3q25.1	4	clarin 1 (CLRN1), transcript variant 5
CNGA1	AR	NM_001142564	4p12	10	cyclic nucleotide-gated channel alpha 1 (CNGA1), transcript variant 1
CNGB1	AR	NM_001297	16q21	33	Homo sapiens cyclic nucleotide-gated channel beta 1 (CNGB1), transcript variant 1
CRB1	AR	NM_201253	1q31.3	12	crumbs homolog 1 (*Drosophila*) (CRB1), transcript variant 1
CRX	AD	NM_000554	19q13.33	4	cone-rod homeobox (CRX)
DHDDS	AR	NM_024887	1p36.11	9	*Homo sapiens* dehydrodolichyl diphosphate synthase (DHDDS), transcript variant 2
EYS	AR	NM_001142800	6q12	43	eyes shut homolog (*Drosophila*) (EYS), transcript variant 1
FAM161A	AR	NM_001201543	2p15	7	family with sequence similarity 161, member A (FAM161A), transcript variant 1
FSCN2	AD	NM_001077182	17q25.3	5	fascin homolog 2, actin-bundling protein, retinal (*Strongylocentrotus purpuratus*) (FSCN2), transcript variant 2
GUCA1B	AD	NM_002098	6p21.1	4	guanylate cyclase activator 1B (retina) (GUCA1B)
IDH3B	AR	NM_006899	20p13	12	isocitrate dehydrogenase 3 (NAD+) beta (IDH3B), nuclear gene encoding mitochondrial protein, transcript variant 1
IMPDH1	AD	NM_000883	7q32.1	17	IMP (inosine 5′-monophosphate) dehydrogenase 1 (IMPDH1), transcript variant 1
IMPG2	AR	NM_016247	3q12.3	19	interphotoreceptor matrix proteoglycan 2 (IMPG2)
KLHL7	AD	NM_001031710	7p15.3	11	kelch-like family member 7 (KLHL7), transcript variant 1
LRAT	AR	NM_004744	4q32.1	3	lecithin retinol acyltransferase (phosphatidylcholine–retinol O-acyltransferase) (LRAT)
MERTK	AR	NM_006343	2q13	19	c-mer proto-oncogene tyrosine kinase (MERTK)
NR2E3	AR/AD	NM_016346	15q23	8	nuclear receptor subfamily 2, group E, member 3 (NR2E3), transcript variant 1
NRL	AD	NM_006177	14q11.2	3	neural retina leucine zipper (NRL)
PDE6A	AR	NM_000440	5q32	22	phosphodiesterase 6A, cGMP-specific, rod, alpha (PDE6A)
PDE6B	AR	NM_000283	4p16.3	22	phosphodiesterase 6B, cGMP-specific, rod, beta (PDE6B), transcript variant 1
PDE6G	AR	NM_002602	17q25.3	4	phosphodiesterase 6G, cGMP-specific, rod, gamma (PDE6G), transcript variant 1
PRCD	AR	NR_033357	17q25.1	5	*Homo sapiens* progressive rod-cone degeneration (PRCD), transcript variant 2
PROM1	AR	NM_006017	4p15.32	27	prominin 1 (PROM1), transcript variant 1
PRPF3	AD	NM_004698	1q21.2-q21.3	16	PRP3 pre-mRNA processing factor 3 homolog (S. cerevisiae) (PRPF3)
PRPF31	AD	NM_015629	19q13.42	14	PRP31 pre-mRNA processing factor 31 homolog (S. cerevisiae) (PRPF31)
PRPF8	AD	NM_006445	17p13.3	43	PRP8 pre-mRNA processing factor 8 homolog (S. cerevisiae) (PRPF8)
PRPH2	AD	NM_000322	6p21.1	3	peripherin 2 (retinal degeneration, slow) (PRPH2)
PRPH2-ROM1	digenic	NM_000327	11q12.3	3	retinal outer segment membrane protein 1 (ROM1)
RBP3	AR	NM_002900	10q11.22	4	retinol binding protein 3, interstitial (RBP3)
RDH12	AR	NM_152443	14q24.1	9	*Homo sapiens* retinol dehydrogenase 12 (all-trans/9-cis/11-cis) (RDH12)
RGR	AR/AD	NM_002921	10q23.1	7	G protein coupled receptor (RGR), transcript variant 1
RHO	AR/AD	NM_000539	3q22.1	5	rhodopsin (RHO)
RLBP1	AR	NM_000326	15q26.1	9	retinaldehyde-binding protein 1 (RLBP1)
RP1	AR/AD	NM_006269	8q12.1	4	retinitis pigmentosa 1 (autosomal dominant) (RP1)
RP2	X-linked	NM_006915	Xp11.23	5	retinitis pigmentosa 2 (X-linked recessive) (RP2)
RP9	AD	NM_203288	7p14.3	6	retinitis pigmentosa 9 (autosomal dominant) (RP9)
RPE65	AR	NM_000329	1p31.3-p31.2	14	retinal pigment epithelium-specific protein 65 kDa (RPE65)
RPGR	X-linked	NM_001034853	Xp11.4	15	retinitis pigmentosa GTPase regulator (RPGR), transcript variant C
SAG	AR	NM_000541	2q37.1	16	S-antigen; retina and pineal gland (arrestin) (SAG)
SEMA4A	AR/AD	NM_001193300	1q22	15	sema domain, immunoglobulin domain (Ig), transmembrane domain (TM) and short cytoplasmic domain, (semaphorin) 4A (SEMA4A), transcript variant 2
SNRNP200	AD	NM_014014	2q11.2	45	small nuclear ribonucleoprotein 200 kDa (U5) (SNRNP200)
SPATA7	AR	NM_018418	14q31.3	12	spermatogenesis-associated 7 (SPATA7), transcript variant 1
TOPORS	AD	NM_005802	9p21.1	3	topoisomerase I binding, arginine/serine-rich, E3 ubiquitin protein ligase (TOPORS), transcript variant 1
TTC8	AR	NM_144596	14q31.3	15	tetratricopeptide repeat domain 8 (TTC8), transcript variant 1
TULP1	AR	NM_003322	6p21.31	15	Tubby-like protein 1 (TULP1)
USH2 / USH2A	AR	NM_206933	1q41	72	Usher syndrome 2A (autosomal recessive, mild) (USH2A), transcript variant 2
ZNF513	AR	NM_144631	2p23.3	4	zinc finger protein 513 (ZNF513), transcript variant 1

### Prioritization of variants

**Fig. 1:**
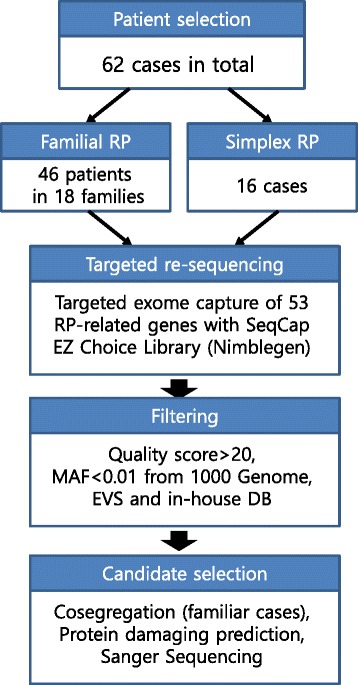
Schematic workflow of the diagnostic application of targeted exome sequencing in familial and simplex retinitis pigmentosa. 62 cases consisting of 46 patients in 18 families and 16 cases in simplex families were recruited, and targeted re-sequencing was performed for 53 RP-related genes. Candidate variants were identified by filtering based on variant quality > 20 and minor allele frequency < 0.01 from 1000 Genome Project (www.1000genomes.org), Exome Variant Server (evs.gs.washington.edu) and our in-house DB consisting of 192 Korean exomes. The variants were finally confirmed by a cosegregation test if familiar cases, *in silico* tools and Sanger sequencing

A flowchart for candidate causal variant detection is shown in Fig. 1. Exonic and splicing variants were first selected if they had an allele frequency below 1 % reported in the NHLBI-ESP 6500 (evs.gs.washington.edu), 1000 Genome Project (www.1000genomes.org), and an in-house database with exomes of 192 Korean individuals. Variants with a quality score below 20 were excluded. Candidate variants were confirmed by Sanger sequencing and co-segregation analysis was performed in cases of familial RP. The potential pathogenicity of variants was categorised into four classes (Table 4). Briefly, Class I included pathogenic variants previously known to cause RP, and Class II variants were expected to cause severe damage to protein structure via frameshift, nonsense, and missense variants, which were likely to cause severe functional change via Polyphen 2 [40], SIFT [41], and MutPred [42]. Class III included variants least likely to be causative and consisted of missense variations that were predicted to be benign or tolerable. All other types of variants were categorised as Class IV. Novel nonsynonymous variants were assumed to be possibly pathogenic if the variant was predicted to be pathogenic by at least two of the three methods. Both probably and possibly damaging mutations were classified as suspected pathogenic variants by Polyphen2. With regard to SIFT, damaging mutations were classified as pathogenic. For Mutpred, a general score higher than 0.5 was categorised as possibly pathogenic.

**Table 4:** Classification of candidate variants in this study

Category	Explanation
Class I	Previously reported pathogenic variants
Class II	Single Nucleotide Variants (SNVs) predicted to cause serious protein deformity by using *in silico* analysis
	Stopgain, and frameshift mutations
Class III	Mutations causing only protein change
Class IV	Other mutations

## Additional files

Additional file 1: Table S1. Quality of sequencing results. **Table S2.** Clinical features of 10 familial RP cases whose strong variants were detected by targeted resequencing. **Table S3.** Clinical features of 7 sporadic RP cases whose strong variants were detected by targeted resequencing. **Table S4.** *In silico* prediction for nonsysnonymous variants. **Figure S1.** Pedigrees of 10 familial cases whose strong variants were detected by targeted re-sequencing. **Figure S2.** Fundus photograph and optical coherence tomography of the interesting cases.
