# Supplementary material for: The diagnostic application of targeted re-sequencing in Korean patients with retinitis pigmentosa
Source: BMC Genomics. 2015 Jul 9;16(1):515. doi: 10.1186/s12864-015-1723-x (PMC4496857; doi:10.1186/s12864-015-1723-x)
Supplement: Additional file 1: Table S1. — Quality of sequencing results. Table S2. Clinical features of 10 familial RP cases whose strong variants were detected by targeted resequencing. Table S3. Clinical features of 7 sporadic RP cases whose strong variants were detected by targeted resequencing. Table S4. In silico prediction for nonsysnonymous variants. Figure S1. Pedigrees of 10 familial cases whose strong variants were detected by targeted re-sequencing. Figure S2. Fundus photograph and optical coherence tomography of the interesting cases. [file 12864_2015_1723_MOESM1_ESM.docx]

# Additional files

**Additional Table 1.** Quality of sequencing results

**Additional Table 2.** Clinical features of 10 familial RP cases whose strong variants were detected by targeted resequencing

**Additional Table 3.** Clinical features of 7 sporadic RP cases whose strong variants were detected by targeted resequencing

**Additional Table 4.** *In silico* prediction for nonsysnonymous variants

**Additional Figure 1.** Pedigrees of 10 familial cases whose strong variants were detected by targeted re-sequencing

**Additional Figure 2.** Fundus photograph and optical coherence tomography of the interesting cases

**Additional Table 1.** Quality data for targeted exome sequencing

| **Reads** | **Mean (± s.d.)** |
| --- | --- |
| Aligned sequence (Mb) | 31.9 (± 5.7) |
| Total reads | 2,887,637 (± 452,554) |
| Aligned paired reads | 2,803,425 (± 433,353) |
| Aligned singleton reads | 9,272 (± 435) |
| % of bases covered to |  |
| ≥x1 | 98.6 (± 0.7) |
| ≥x10 | 96.5 (± 1.6) |
| ≥x25 | 92.9 (± 3.6) |
| ≥x50 | 85.0 (± 7.7) |
| ≥x75 | 75.0 (± 12.0) |
| ≥x100 | 64.1 (± 14.6) |

**Additional Table 2.** Clinical features of 10 familial RP cases whose variants were detected by targeted resequencing

| Family | Gene | Sex | Age (y) | Symptom onset age (y) | Lens | VA (OD) | VA (OS) | ERG | OCT | GVF |
| --- | --- | --- | --- | --- | --- | --- | --- | --- | --- | --- |
| F03 | RP1 | M | 21 |  |  | 0.7 | 0.8 | extinguished | PR 1.8/1.6 | mild superior field constriction |
| F04 | RP2 | M | 6 | 4 |  | 0.1 | 0.02 | only low response at 30 Hz flicker | PR -/- | mildly constricted |
| F06 | RP1 | M | 40 | 35 |  | 1.2 | 1.2 | extinguished | PR 2.9/3 | 10°~20° |
| F07 | PRPF31 | M | 33 | 10 |  | 0.7 | 0.8 | extinguished | PR 1.1/1 | 10° and temporal island |
| F09 | RHO | F | 49 | 7 | Cat | LP | LP | extinguished | PR -/- | (-) |
| F10 | KLHL7 | F | 34 | 23 |  | 0.6 | 0.6 | extinguished rod response, markedly reduced cone response | PR 1.2/1.3, ERM | 10° |
| F12 | RP2 | M | 52 | 4 | Cat | LP | HM | extinguished | PR -/-, ERM |  |
| F13 | RHO | M | 52 | 16 | PCL | HM | NLP |  | PR -/- |  |
| XF1 | TOPORS | F | 33 | 13 |  | 0.5 | 0.3 |  | PR 1.2, CME |  |
| XF3 | PRPF31 | M | 46 | 8 | Cat | 0.4 | 0.3 | extinguished | PR 0.9 | 5° |

Only the clinical features of the proband (indexed patients of the families) were described.

PCL: cataract operation was done; Cat: lens opacity (cataract)

VA: best corrected visual acuity (Snellen); OD: right eye; OS: left eye

NLP: no light perception; LP: light perception; HM: hand movement; FC: counting finger

ERG: standard electro-retinogram, ISCEV protocol

OCT: optical coherence tomography

PR: preserved photoreceptor inner and outer segment junction in the horizontal macular scan (OD/OS)

ERM: epiretinal membrane; CME: cystoid macular edema

GVF: Goldmann visual field test

The visual field test results between both eyes were nearly identical.

**Additional Table 3.** Clinical features of 7 sporadic RP cases whose variants were detected by targeted resequencing

| Patient | Gene | Sex | Age (y) | Symptom onset age (y) | Cataract | VA (OD) | VA (OS) | | ERG | OCT | GVF |
| --- | --- | --- | --- | --- | --- | --- | --- | --- | --- | --- | --- |
| 430 | PRPF31 | M | 42 |  |  | 1 | 0.9 | | mildly decreased rod response | PR 5/5.6 | paracentral scotoma |
| 432 | PRPH2 | M | 28 | 6 | cat | NLP | 0.4 | | extinguished |  | OD) (-) OS) 5° |
| 436 | PDE6B | F | 24 | 13 | cat | 1.2 | 1 | |  |  |  |
| 438 | USH2A | M | 58 | 15 | cat | FC | FC | | extinguished | PR -/- | 5° |
| 439 | EYS | M | 57 | 15 | cat | 0.6 | 0.5 | |  |  | 5°~10° |
| 440 | EYS | F | 37 | 30 |  | 0.4 | 0.5 | | extinguished | PR 3/2.6 | 5~10° |
| 445 | PDE6B | M | 60 | 3 | PCL | 0.2 | 0.02 | |  | PR -/- | <5° |
| PCL: cataract operation was done; Cat: lens opacity(cataract)  VA: best corrected visual acuity (Snellen); OD: right eye; OS: left eye  NLP: no light perception; LP: light perception; HM: hand movement; FC: counting finger  ERG: standard electro-retinogram, ISCEV protocol  OCT: optical coherence tomography  PR: preserved photoreceptor inner and outer segment junction in the horizontal macular scan (OD/OS)  ERM: epiretinal membrane; CME: cystoid macular edema  GVF: Goldmann visual field test  The visual field test result between both eyes were nearly identical in all cases, except in patient 432. | | | | | | | |  |  |  |  |

**Additional Table 4.** *In silico* prediction for nonsysnonymous variants

| No | Gene | nucleotide | amino acid | Reference | Class | Polyphen2 | SIFT | MutPred |
| --- | --- | --- | --- | --- | --- | --- | --- | --- |
| F04 | RP2 | c.340T>C | p.C114R | novel | Ⅱ | Prob (1.000) | Dam | 0.904 (0.0051) |
| F09 | RHO | c.1040C>T | p.P347L | rs29001566 | I | Prob (1.000) | Dam | 0.811 (0.0462) |
| F10 | KLHL7 | c.458C>T | p.A153V | rs137853113, 11 Wen | I | Prob (0.993) | Dam | 0.943 |
| F13 | RHO | c.533A>G | p.Y178C | rs104893776 | I | Prob (1.000) | Dam | 0.917 |
| XF4, 450 | USH2A | c.10246T>G | p.C3416G | 13 Huang | I | Prob (0.999) | Dam | 0.448 |
| 430 | PRPF31 | c.310G>A | p.E104K | Novel | Ⅱ | Prob (0.967) | Dam | 0.745 (0.0198) |
| 432 | PRPH2 | c.380A>G | p.E127G | Novel | Ⅱ | Ben (0.121) | Dam | 0.54 |
| 435 | EYS | c.7394C>G | p.T2465S | rs145184183 | Ⅱ | Prob (0.990) | Tol | 0.614 (0.0349) |
| 436, 445 | PDE6B | c.832C>T | p.H278Y | rs121918581 | Ι | Prob (0.991) | Dam | 0.929 |
| 438 | USH2A | c.8885T>G | p.L2962R | Novel | Ⅱ | Prob (1.000) | Dam | 0.752 (0.0126) |
| 440 | EYS | c.6557G>A | p.G2186E | 10 Littink | Ι | Poss (0.946) | Dam | 0.926 |
| 445 | PDE6B | c.767T>A | p.I256N | Novel | Ⅱ | Prob (0.981) | Dam | 0.793 |
| 450 | USH2A | c.6683T>A | p.V2228E | rs117461552 | Ⅱ | Poss (0.868) | Dam | 0.437 |

No: family or patient identifier

Class: Classification of candidate variants in this study described in Table 1

Prob: probably damaging; Poss: possibly damaging; Ben: benign

Dam: damaging; Tol: tolerable


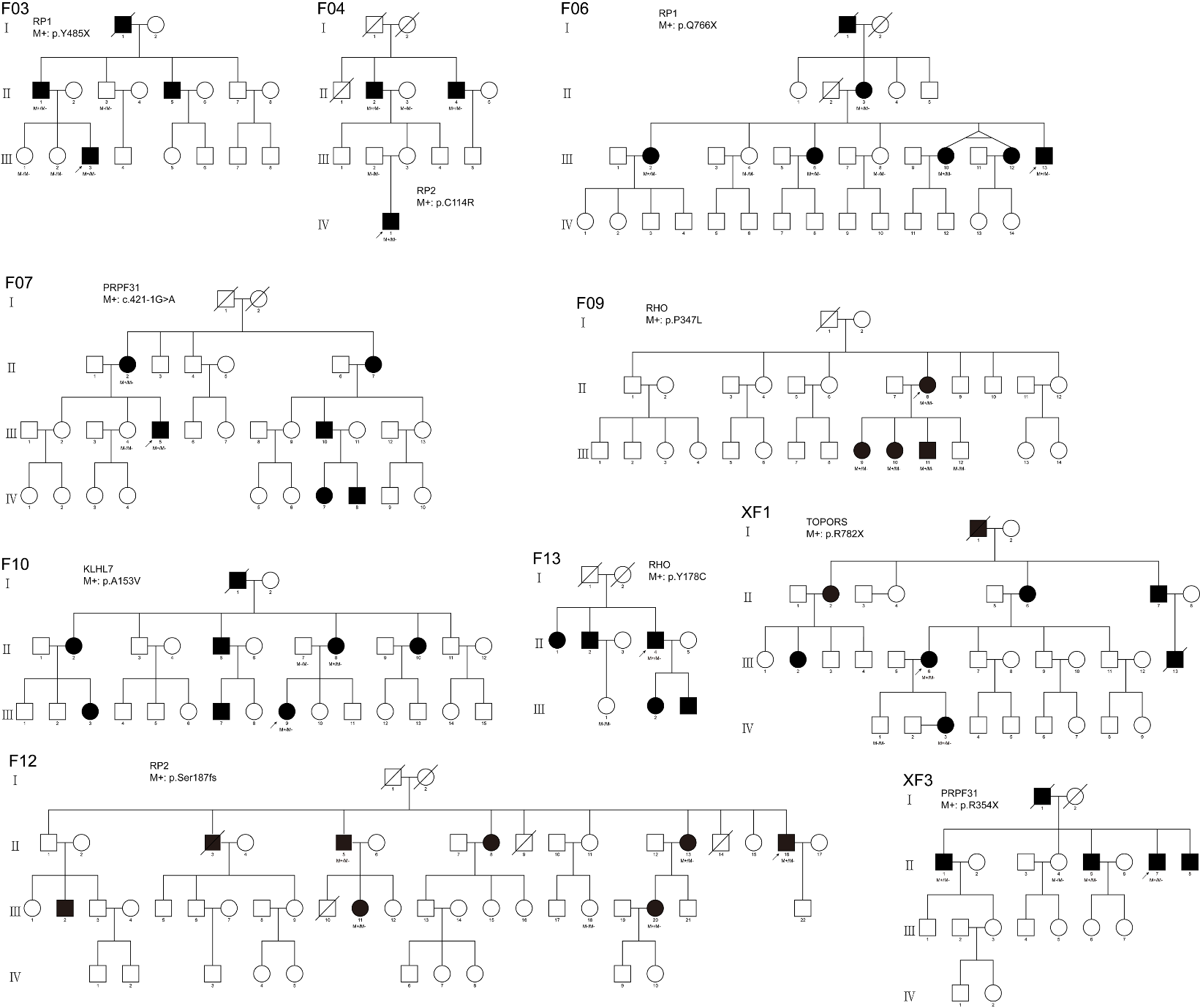


# Additional Figure 1. Pedigrees of 10 familial cases whose strong variants were detected by targeted re-sequencing


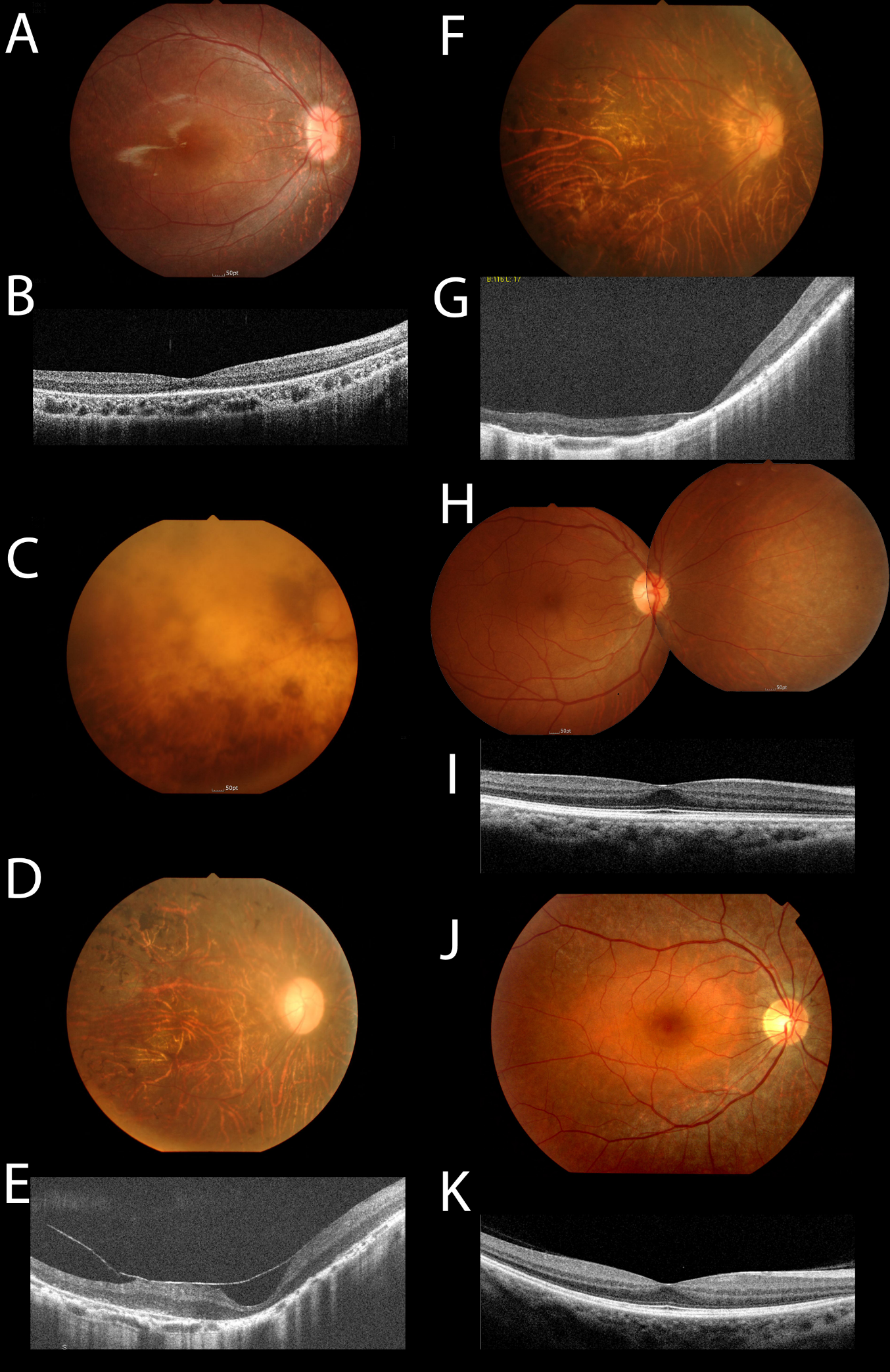


# Additional figure 2. Fundus photograph and optical coherence tomography of the interesting cases

(A-C) F04 family whose candidate variants was p.C114R in *RP2*, (A) Fundus photograph and (B) OCT of IV-1 patient. He was a 6-year-old and his visual acuity was 20/200 (OD) and 20/1000 (OS). Although fundus appearance looks not so degenerated, the photoreceptor inner segment and outer segment junction was not detectable using OCT. (C) Fundus photograph of Ⅱ-2 patient who is grandfather of IV-1 patient. This showed severely degenerated retina. (D-G) F12 family whose candidate variant was p.Ser187fs in *RP2*. (D) Fundus photograph and (E) OCT of Ⅱ-16 patient who is hemizygote male. (F) Fundus photograph and (G) OCT of Ⅱ-13 patient who is heterozygote female. Both Ⅱ-16 and Ⅱ-13 showed severely degenerated retina compatible with RP. Other female member of this family showed variable expression of RP. (H and I) F06 family whose candidate variant is p.Q766X in *RP1*. (H) Fundus photograph and (I) OCT of Ⅲ-6 patient. She was 49 years old and had normal vision. Fundal appearance looks almost normal except pigmentary retinal deposit seen at nasal peripheral portion. OCT scan showed intact macular structure. (J and K) Sporadic patient 430. Candidate variant was p.E104K in *PRPF31*. He was 42 years old. He had normal vision. Retinal degeneration is seen outside the arcade at fundus photograph. The photoreceptor inner segment and outer segment junction damage is only seen at periphery using OCT.
